# Focused Update on Clinical Testing of Otolith Organs

**DOI:** 10.3390/audiolres14040051

**Published:** 2024-07-02

**Authors:** Stefan C. A. Hegemann, Anand Kumar Bery, Amir Kheradmand

**Affiliations:** 1 Balance Clinic Zurich, Nüschelerstrasse 49, CH-8001 Zurich, Switzerland; 2 Faculty of Medicine, University of Zurich, CH-8005 Zurich, Switzerland; 3 Department of Neurology, School of Medicine, The Johns Hopkins University, Baltimore, MD 21287, USA; abery1@jh.edu (A.K.B.); akherad@jhu.edu (A.K.); 4 Department of Otolaryngology-Head and Neck Surgery, School of Medicine, The Johns Hopkins University, Baltimore, MD 21287, USA; 5 Department of Neuroscience, School of Medicine, The Johns Hopkins University, Baltimore, MD 21287, USA

**Keywords:** otolith receptors, testing, otoconia, VEMPs, SVV, SVH, binocular cyclorotation, video ocular counter roll

## Abstract

Sensing gravity through the otolith receptors is crucial for bipedal stability and gait. The overall contribution of the otolith organs to eye movements, postural control, and perceptual functions is the basis for clinical testing of otolith function. With such a wide range of contributions, it is important to recognize that the functional outcomes of these tests may vary depending on the specific method employed to stimulate the hair cells. In this article, we review common methods used for clinical evaluation of otolith function and discuss how different aspects of physiology may affect the functional measurements in these tests. We compare the properties and performance of various clinical tests with an emphasis on the newly developed video ocular counter roll (vOCR), measurement of ocular torsion on fundus photography, and subjective visual vertical or horizontal (SVV/SVH) testing.

## 1. Introduction

### The Structure and Function of the Otolithic Receptors 

A normally functioning vestibular system is critical for spatial orientation, balance, and steady gaze while the head is in motion. Unlike the semicircular canals that detect angular acceleration, the otolith organs detect linear displacement with translational movements of the head or tilting against the pull of gravity. This is a key function for sensing gravity as a frame of reference to maintain spatial orientation while interacting with the environment [1]. The inputs from otolith organs also control eye movement and posture with changes in the head position. These diverse contributions form the basis for clinical testing of otolith function, which may vary in measurement outcomes. This variability depends on the method of stimulation as well as the structure and physiology of otolith organs, and how they may collectively contribute to functional measurements in each test. Here, we first describe these key physiological aspects pertinent to the measurement of otolith function and then focus on common clinical evaluations for testing otolith function. 

The orientation of the sensory epithelium or the macula within each otolith organ allows for the detection of motion within the same plane. The utricular macula is aligned horizontally, and the saccular macula is aligned vertically. The utricular macula has stronger projections to the ocular motor system, and the saccular macula has stronger projections to the vestibulospinal system [2]. The macula of each otolith organ consists of a mix of type I and II hair cell receptors. Type 1 receptors are mainly located in the striola or central zone of the macula and are connected to afferents with irregular resting discharge sensitive to sound and vibration [2]. Type II receptors are located on either side of the central zone with opposite polarities (i.e., hair cell deflections in opposite directions) and are connected to afferents with regular discharges sensitive to low-frequency linear acceleration. This different pattern of stimulation is related to the morphology of the hair cell receptors. Type I receptors have short hair bundles that do not reach the gelatinous layer covering the hair cells, but type II receptors have long projections protruding into the gelatinous layer covered by the crystals of dense otoconia [3]. Because of their short projections, type I receptors are sensitive to higher-frequency stimulation like sound and vibrations, while type II receptors are more responsive to low-frequency linear acceleration or static tilt against the constant pull of gravity because of the high density of otoconia on the hair cell projections (for review, see [2,3]).

The unique anatomical characteristics of hair cells highlight the significance of otoconia in facilitating the function of type II afferents in particular. These afferents maintain a consistent baseline activity and are sensitive to gravity or low-frequency linear accelerations, forming what is known as the static or sustained otoconial system [3]. The striolar type 1 hair cells, however, lack otoconia and with their irregular afferents are referred to as the transient system. A good example of a test using the sustained system is the response to maintained head tilt. Pathologies that affect otoconial mass such as head trauma or age-related degeneration may also affect the sustained pathway and reduce deflection of hair cell bundles by the pull of gravity [4,5,6]. Studies suggest a loss in otoconial mass of about 40% in the utricle and 70% in the saccule at the age of 80 [7,8,9]. No difference, however, is found between type I and type II hair cells on human maculae utriculi or sacculi with aging and both are reduced by about 25% [10]. According to Rauch et al., the ratio of type I to type II hair cells is about 1.3 in the macula based on 67 temporal bones from 49 individuals (age range from birth to 100 y/o) [10]. Similarly, Gopen et al., found that the ratio of type I to type II hair cells was about 1.7 in few subjects with an age range of 42 to 96 y/o [11]. Unlike hair cells in the macula receptors, hair cells in the cristae of the semicircular canals and the cochlea show a reduction of about 40% with aging [10]. In humans, no specific pathology is associated with the exclusive loss in type II hair cells, although pronounced loss in type II hair cells has been reported in Meniere’s disease and following irradiation of the inner ear in animal models [12,13]. The key role of otoconia in vestibular physiology is also shown with mouse models of otoconial loss [14,15]. These animals have lifelong balance problems despite having normal hair cell morphology.

Given the distinct populations of hair cells across the macula receptors that respond differently to stimulus type, onset, and duration, these structural and functional implications must be considered for clinical testing of otolith function. In the next sections, we review common methods used for the clinical evaluation of otolith function and discuss how different aspects of otolith physiology may affect functional measurements in these tests. In this context, we compare measurement outcomes from different clinical tests.

## 2. Clinical Evaluation of the Otolith Organs

Common clinical tests for the evaluation of otolith function include (i) the vestibular evoked myogenic potentials (VEMP) [16], (ii) the video ocular counter roll (vOCR) [17], (iii) measurement of the torsional eye position or ocular torsion using fundus photography [18], and (iv) the tests of subjective visual vertical (SVV) or horizontal (SVH) for the assessment of perceived direction of gravity [19] (Table 1). Other less-common tests are off-axis rotation (OVAR) [20,21] and translational VOR (tVOR) [22].

As supported by recordings from type I hair cells, VEMP testing is based on the activation of hair cells by high-frequency air-conducted sound or bone-conducted vibration [23]. VEMP responses from ocular muscles or oVEMP are used as a measure of utricular function, and responses from cervical muscles or cVEMP are used as a measure of saccular function. The common stimulus for VEMP testing is either sound or vibration at a frequency of 500 Hz, which results in selective activation of irregular neurons from the striola. At this frequency (500 Hz), the irregular afferents within the semicircular canals are not usually stimulated by sound or vibration [23]. For cVEMP testing, the patient is instructed to contract their sternocleidomastoid muscle while the stimulus is played to record inhibitory evoked responses using surface electrodes from the same side of the neck. A characteristic response is a biphasic waveform with a latency of the peaks at 13 and 23 milliseconds. Typically, a threshold of sound intensity that can produce the characteristic waveform is obtained. Similar stimuli can be used for oVEMP testing in which a crossed excitatory response is recorded from the contralateral inferior oblique muscle. The response is also biphasic with a first negative waveform at 10 milliseconds.

**Figure 1:**
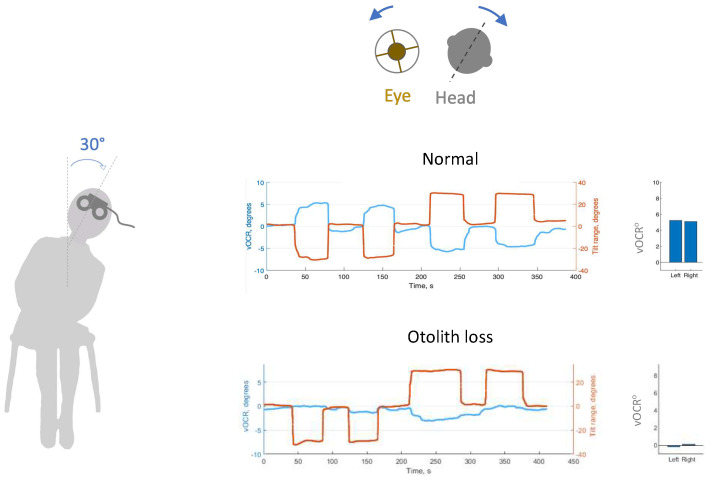
The video ocular counter roll (vOCR) is a clinical test of ocular-otolith function. The tilt maneuver is a 30° lateral tilt of the lateral tilt of the head and trunk en bloc. During sustained head tilt, the eyes maintain roll in the opposite direction driven primarily by inputs from the utricle. In a normal subject, vOCR is greater than 4^o^ (blue) with 30^o^ head tilt in (red); i.e., gain of larger than 0.15. In contrast, a patient with otolith loss shows a significantly reduced vOCR.

Because of the high density of otoconia, type II receptors are more responsive to low-frequency linear acceleration or static tilt against the constant pull of gravity. Clinical tests of otolith function that involve static head tilt exploit this property. Tilting the head results in a torsional vestibular ocular reflex (VOR) or ocular counter roll (OCR), during which the eyes roll in the opposite direction of the head tilt. During the movement of the head, a combination of otolith and semicircular canal stimulation results in a torsional nystagmus with the slow phase in the opposite direction of the head tilt. During the static head tilt, however, the OCR is driven primarily by inputs from the utricle [24]. The static OCR has a normal gain (eye position divided by head position) of about 0.15 that can be measured using a video oculographic method known as the vOCR [17] (Figure 1).

While the vOCR is a test of torsional VOR, measurement of ocular torsion when the head is upright can evaluate the otolith-ocular tone balance [25]. In humans, ocular torsion is the major component of vestibular response during lateral head tilt known as the physiological ocular tilt reaction. This involves rolling of the eyes in the opposite direction of the head tilt to realign with the direction of gravity. Consider a motorcycle rider going around a tight bend to the right. The lateral tilt of the body excites the right utricle resulting in the following: (1) a head tilt to the left, (2) an ocular counter roll with the top pole of each eye rotated toward left, and (3) a compensatory skew deviation with upward movement of the lowermost right eye and downward movement of the uppermost left eye. In cases where a pathology leads to utricular tone imbalance, an ocular tilt response may also emerge even with the head upright. This pathological or ‘false’ ocular tilt response consists of the same three components including a lateral head tilt, skew deviation, and pathological ocular roll or torsional misalignment of the eyes with the top poles rotating toward the side of the lower eye.

The otolith-ocular pathway decussates within the brainstem, and as a result, lesions of the same pathway at different levels can lead to different directions of pathological ocular roll. In lesions of the utricle and lower brainstem (i.e., prior to decussation), the top pole of each eye is rotated towards the ipsilateral side. Conversely, if the pathway is lesioned in the upper brainstem (i.e., after decussation), the top pole of each eye is rotated toward the contralateral side [26,27,28]. This feature of the pathway is outlined in greater detail in the section on SVV.

The effect of isolated loss in utricular function is also shown in animal models. Unilateral loss in utricular function in guinea pigs resulted in strong postural changes at the acute stage with head roll tilt toward the affected side [29]. These responses are similar to the ocular tilt reaction with complete unilateral vestibular loss in humans, gradually diminishing with time as recovery and vestibular compensation occur [30].

**Figure 2:**
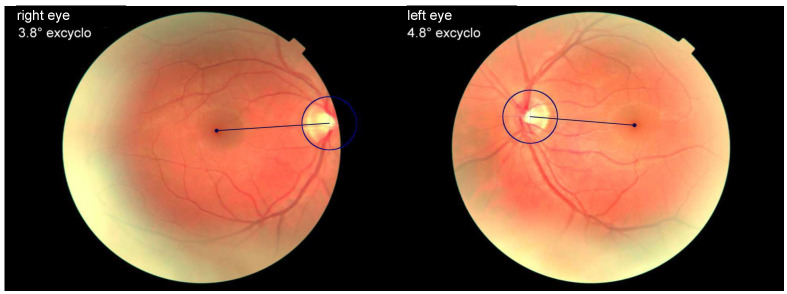
Fundus photography showing normal binocular cyclorotation. Excyclotropy left eye 4.8°, excyclotropy right eye 3.8°.

**Figure 3:**
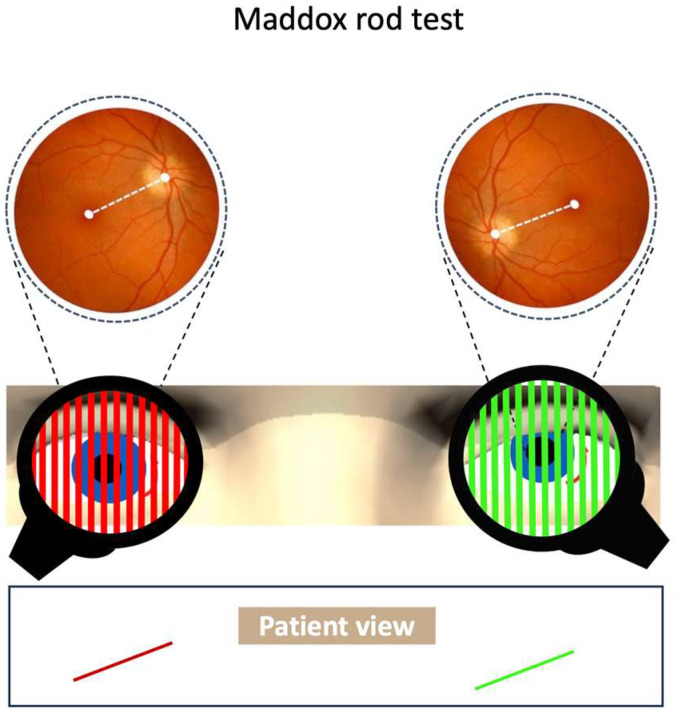
In pathological ocular roll, there is no or little torsional disparity in fundus photos. The dashed line shows the orientation of the fundus in each eye as the disc-foveal line. Patient view shows the relative position of the images from each eye with the double Maddox rod test.

The pathological ocular roll can be assessed on fundus photos by measuring the angle between the optic disc and fovea (Figure 2) [31]. The normative value for the disc foveal angle is within a range of 5–7° [32]. Another method for evaluation of torsional alignment of the eyes uses the double Maddox rod (Figure 3) [25]. This test can be easily performed at bedside for the assessment of otolith-ocular tone balance. The Maddox rod is a filter that converts a source of light into a line when held in front of the eye. The visual line orientation seen with each eye can be used to assess torsional alignment of the eyes. The degree of torsional misalignment with the pathological ocular roll is fairly uniform in both eyes (i.e., there is no difference between the two eyes, and the deviation is comitant).

**Figure 4:**
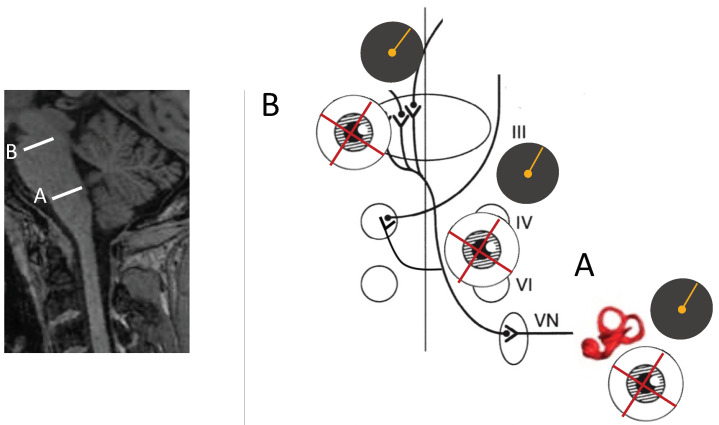
Both SVV (orange line) and pathological ocular roll (red cross) show deviations in the same direction. Pathologies affecting the labyrinth or the lower brainstem (medulla and lower pons/A) result in SVV and ocular roll deviations towards the lesion side, whereas pathologies within the higher brainstem (higher pons and midbrain/B) result in deviations away from the lesion side. VN: vestibular nucleus, VI: sixth cranial nerve, IV: 4th cranial nerve, III: 3rd cranial nerve.

The perceptual consequence of the ocular counter roll is shift in the subjective visual vertical (SVV), measured as the angle between perceptual and gravitational (true) vertical. Consequently, SVV should be another sensitive sign of a disturbance in the otolith-ocular pathways. Measurement of SVV can be done reliably and quickly at bedside using the bucket test [33]. In this test, the subject places their head inside a bucket to align a straight line visible on the bottom of a bucket to vertical orientation. Visual cues are removed as the subject’s head is inside the bucket, and proprioceptive cues are removed as the bucket is randomly rotated to the right or left by the examiner. SVV can also be measured by presenting a visual line on portable devices in a completely dark room. In the upright position, normal individuals can position a visual line in an otherwise completely dark room within two degrees of true vertical [34,35]. Patients with acute peripheral or central vestibular lesions, however, have a perceived vertical that deviates from the true vertical by several degrees. As a topographic rule, lesions affecting the labyrinth or the lower brainstem (caudal pons and rostral medulla) cause ipsilateral SVV deviations, and lesions in the higher brainstem (rostral pons and midbrain) cause contralateral SVV deviations (Figure 4) [36]. As a measure of spatial orientation in reference to gravity, SVV measurement is also affected by other sensory modalities including vision and body proprioception. Because of such multisensory contributions, SVV deviation can be dissociated from VOR [37] and thus should not be considered as a specific test of utricular function. Therefore, while SVV deviation can be caused by vestibular imbalance, pathologies affecting other sensory modalities and their sensory integration may also lead to SVV abnormalities [19,38].

## 3. Comparisons of Clinical Tests of Otolith Function

When comparing different tests of otolith function, one must consider the difference between subjective and objective measurement methods, how they could be specifically affected by multisensory or otolith-specific contributions, and how each test may vary in the stimulation of hair cell populations.

Studies that have compared SVV and VEMP measurements have shown mixed results without any clear association between VEMP and perception of gravity in pathologies such as Meniere’s disease or vestibular neuritis, with more discrepancies found between acute and chronic patients [39,40,41]. These findings are in line with the multisensory nature of SVV compared to VEMP as a more specific and objective measure of otolith function [42]. Similar dissociation is found between ocular torsion and SVV measurements in patients with vestibular pathologies [41,43]. The SVV deviation normalized in most patients within few months of recovery, whereas ocular torsion remained abnormal even a year later [40], suggesting some role for central compensation in vertical perception.

When analyzed separately, oVEMP and ocular torsion abnormalities were present in about 70% of cases with vestibular neuritis [39,41,44,45,46]. However, when measured together in the same group of patients, ocular torsion and oVEMP abnormalities overlapped in about 50% of cases [43]. The oVEMP alone was abnormal in 40% of cases, and ocular torsion alone was abnormal alone in about 10% of cases [43]. This discrepancy could be related to different aspects of utricular function captured differently by oVEMP and ocular torsion measurements. While oVEMP responses mainly reflect the activity of type I hair cells that are sensitive to sound or vibration on the utricular macula, ocular torsion reflects the overall balance of utricular inputs with the torsional deviation pointing towards the weaker side.

The oVEMP response to sound or vibration is primarily transmitted by irregular utricular afferents [3,47,48]. These striolar type 1 hair cells and their irregular afferents are called the transient system, which may remain unaffected in cases with loss of otoconia. While the transient system is preferentially reacting to dynamic stimulation, i.e., high-frequency linear acceleration, the sustained system reacts more to stimulation by lower frequencies. As a constant, low-frequency acceleration, the pull of gravity is transmitted by the sustained system. Since the vOCR response is generated by the pull of gravity on the utricular macula, it may primarily reflect the function of type II hair cells, whereas the VEMP response would reflect the function of the type I hair cells sensitive to sound and vibration. Such a distinction could be valuable in examining differential involvement of the transient and sustained pathways of utricular afferents. Overall, as a test of utricular–ocular function, vOCR demonstrates a sensitivity level comparable to VEMP in detecting vestibular loss [49]. However, unlike VEMP responses, vOCR deficits often recover after vestibular injury [17,30,50]. With acute vestibular loss, vOCR is mainly reduced on the affected side, but there is a symmetrical reduction on both sides with chronic vestibular loss [49,50]. In line with this finding, the largest vOCR deficit is observed in patients with bilateral vestibular loss [49].

## 4. Conclusions

The overall contribution of otolith receptors to eye movement, postural control, and perceptual functions is the basis for clinical testing of otolith function. With such broad contributions, it is important to recognize that the functional outcomes of these tests may vary depending on the specific method employed to stimulate the hair cells or measure the otolith-ocular tone balance. Within the otolithic maculae, different neural afferents have different selectivity; type I afferents are sensitive to sound and vibration, while type II afferents are tuned to low-frequency linear acceleration and static head tilt. The type I hair cells and their irregular afferents are called the transient system, and type II hair cells with their constant baseline activity are called the sustained system. These unique anatomical characteristics of hair cells highlight the significance of otoconia in facilitating the function of type II afferents in sensing the pull of gravity.

Common clinical tests for the evaluation of otolith function include VEMP, vOCR, assessment of ocular torsion using fundus photography, and SVV as a measure of perceived vertical orientation. VEMP is an objective test that exploits a low-level reflex, mostly representing the function of type I hair cells. The new vOCR test assesses the physiologic ocular counter roll in response to a static head tilt, which is mostly consistent with the stimulation of type II hair cells and otoconial function. While the vOCR is a test of torsional VOR, measurement of ocular torsion with the head upright can evaluate otolith-ocular tone balance. The perceptual consequence of the ocular counter roll is shift of perceived vertical that can be measured as the SVV error. When comparing these various tests of otolith function, one must consider the difference between subjective and objective measurement methods, how they may vary in stimulation of hair cell populations, and how the results may be affected by multisensory or otolith-specific contributions.

## Figures and Tables

**Table 1:** Clinical tests of otolith-ocular function.

Test	Method	Stimulus Pathway	Measurement	Interpretation
VEMP	Sound-/vibration-induced myogenic potential	Type 1 hair cells (transient system)	Biphasic wave (oVEMP/utricle and cVEMP/saccule)	Objective
vOCR	VOG measure (30° lateral head tilt)	Type 2 hair cells (sustained system)	Ocular counter roll	Objective
Fundus photograph	Ocular torsion alignment	N/A *	Disc foveal angle **	Objective
Maddox rod	Ocular torsion alignment	N/A *	Perceived line angle **	Subjective
SVV	Perceived vertical	N/A *	Perceived vertical angle	Subjective

* Measure of otolith-ocular tone balance. ** Both eyes roll towards the ipsilateral ear in a peripheral or lower brainstem lesion and towards the contralateral ear in a higher brainstem lesion; e.g., in a left peripheral vestibular or a right rostral brainstem lesion, the top pole of each eye will rotate towards the left ear (excyclotorsion of the left eye and incyclotorsion of the right eye). VEMP: Vestibular Evoked Myogenic Potentials; vOCR: Video Ocular Counter Roll; SVV: Subjective Visual Vertical.
